# Links Between Behavior Change Techniques and Mechanisms of Action: An Expert Consensus Study

**DOI:** 10.1093/abm/kay082

**Published:** 2018-11-19

**Authors:** Lauren E Connell, Rachel N Carey, Marijn de Bruin, Alexander J Rothman, Marie Johnston, Michael P Kelly, Susan Michie

**Affiliations:** 1 Centre for Behaviour Change, University College London, London; 2 Department of Kinesiology, University of Rhode Island, Kingston; 3 Aberdeen Health Psychology Group, Institute of Applied Health Sciences, University of Aberdeen, Health Sciences Building, Foresterhill, Aberdeen; 4 Department of Psychology, University of Minnesota, Minneapolis; 5 Primary Care Unit, Institute of Public Health, University of Cambridge, Cambridge

**Keywords:** Behavior change, Theory, Methodology, Behavior change technique, Mechanism of action, Expert consensus

## Abstract

**Background:**

Understanding the mechanisms through which behavior change techniques (BCTs) can modify behavior is important for the development and evaluation of effective behavioral interventions. To advance the field, we require a shared knowledge of the mechanisms of action (MoAs) through which BCTs may operate when influencing behavior.

**Purpose:**

To elicit expert consensus on links between BCTs and MoAs.

**Methods:**

In a modified Nominal Group Technique study, 105 international behavior change experts rated, discussed, and rerated links between 61 frequently used BCTs and 26 MoAs. The criterion for consensus was that at least 80 per cent of experts reached agreement about a link. Heat maps were used to present the data relating to all possible links.

**Results:**

Of 1,586 possible links (61 BCTs × 26 MoAs), 51 of 61 (83.6 per cent) BCTs had a definite link to one or more MoAs (mean [*SD*] = 1.44 [0.96], range *=* 1–4), and 20 of 26 (76.9 per cent) MoAs had a definite link to one or more BCTs (mean [*SD*] = 3.27 [2.91], range = 9). Ninety (5.7 per cent) were identified as “definite” links, 464 (29.2 per cent) as “definitely not” links, and 1,032 (65.1 per cent) as “possible” or “unsure” links. No “definite” links were identified for 10 BCTs (e.g., “Action Planning” and “Behavioural Substitution”) and for six MoAs (e.g., “Needs” and “Optimism”).

**Conclusions:**

The matrix of links between BCTs and MoAs provides a basis for those developing and synthesizing behavioral interventions. These links also provide a framework for specifying empirical tests in future studies.

## Teaser

Experts in behaviour change science agreed on the processes through which a number of different techniques are hypothesized to change behaviour. This information will help people design behaviour change interventions and guide efforts to determine how these interventions work.

## Introduction

Behavior change interventions have the potential to improve health, reduce premature mortality [1], disability [2], and health care expenditures [3]. To achieve this, effective interventions that lead to sustained behavior change are needed [4]. Given the complexity of behavior change interventions, it is important to identify the potentially active ingredients within an intervention (i.e., behavior change techniques [BCTs]), the processes through which behavior change occurs (i.e., the “mechanisms of action” [MoAs]), and the links between BCTs and MoAs. The potentially active ingredients (BCTs) in an intervention are those aspects of an intervention which produce a change in behavio. The BCTs produce a change in behavior by acting upon a process (e.g., a psychological, physical, or social process) which changes as a result of the active ingredient, this change is what catalyzes a change in behavior. We define MoAs as processes which influence behavior. We define a BCT-MoA link as a pathway through which behavior change occurs, via a specific BCT producing change in a specific MoA. An improved understanding of the links between BCTs and MoAs can facilitate the development of more effective interventions and improve the ability to explain how effective interventions bring about change.

Behavioral science has made substantial progress in harmonizing and standardizing the reporting of interventions [5–9] and their theoretical underpinnings [10–12], which facilitates communication across disciplines and supports intervention replication and implementation. Intervention reporting has been enhanced through the development of methods to describe potentially active ingredients within interventions systematically [7, 13], thereby facilitating knowledge accumulation across different interventions. The 93-item Behavior Change Technique v1 Taxonomy, for example, was developed with contributions from a large international network of behavior change experts, and is a formal and standardized classification system of labels and definitions of these intervention techniques (i.e., which potentially active ingredients are delivered within an intervention) [7]. To date, the BCT v1 Taxonomy has been used across a wide range of behavioral domains to specify content for intervention reports, to aid in intervention design, and to synthesize information across intervention evaluations (see http://www.bct-taxonomy.com/interventions; last accessed on October 30, 2018. for a searchable database of over 350 articles reporting interventions coded by BCTs).

The BCT v1 Taxonomy provides a shared language with which to describe intervention content (i.e., BCTs); however, it does not directly specify which MoAs these BCTs target. The importance of understanding the links between MoAs and BCTs is highlighted in frameworks for the development of behavior change interventions (e.g., Intervention Mapping [9], Precede-Proceed [14], Behavior Change Wheel [15]). Identifying specific links is important in developing interventions and understanding the process through which behavior change may occur, as emphasized by the Cochrane Collaboration’s Effective Practice and Organization of Care (EPOC) Group [16], and from The National Institute for Health and Care Excellence’s (NICE) Public Health guidelines in the UK [17, 18]. In the USA, the *Science of Behavior Change* initiative has also highlighted this need and is building knowledge in this area by experimentally testing methods for changing specified MoAs (see https://commonfund.nih.gov/behaviorchange/index; last accessed on October 30, 2018).

There are direct ways of generating evidence on how BCTs and MoAs are linked, namely, experimental studies and meta-analyses thereof [19]. However, currently available evidence is insufficient to do that for a large number of BCTs and MoAs. There are also several indirect approaches that allow for exploring how a larger set of BCTs and MoAs are linked. One way to infer how BCTs are related to MoAs is to investigate links between BCTs and MoAs explicitly hypothesized in the published intervention literature (see Carey et al. 2018). Although the published literature provides valuable information, it is limited by what research has been funded, which findings have been published, and what researchers choose to report. A complementary source of evidence is the current thinking of international experts in behavior change. This source of information encompasses the existing hypotheses of experts in the field, which are unhindered by publication and funding constraints, yet also informed by existing theory and evidence, including evidence from current research. One method of examining experts’ hypotheses is through expert consensus methodology.

Expert consensus methods can be used to facilitate the development of research questions, solutions to existing problems, and priorities for action [20]. They enable differing ideas on topics of mutual interest to be discussed, reported, and organized, with a view to establishing areas of consensus and priorities for further investigation. Participation in this approach also tends to foster the participants’ ownership of the resulting research and thus increases the likelihood of changing future practice and research [21].

This study is one of four in a program of research to develop and test a methodology for linking BCTs to MoAs (see Ref. 22 for the protocol). The aim of the current study is to develop—based on expert consensus—an overview of the mechanisms through which BCTs might alter behavior. Specific questions are as follows: (a) Through which MoAs do experts agree BCTs influence behavior? (b) Through which MoAs do experts agree BCTs do not influence behavior? (c) How specific are the mechanisms through which BCTs have an effect, that is, do experts agree BCTs influence behavior through one MoA or that they can influence behavior through multiple MoAs? Subsidiary questions are as follows: (d) About which links between MoAs and BCTs do experts disagree? (e) Can all BCTs be linked to at least one MoA? (f) Can all MoAs be linked to at least one BCT?

## Methods

### Design

Expert consensus about links between BCTs and MoAs were investigated using a formal consensus method drawing on Nominal Group Technique (NGT) [23] in three rounds: (i) an initial rating round, (ii) a discussion round, and (iii) a final rating round.

### Participants

Participants were experts with experience in designing, evaluating, and/or synthesizing evidence about theory-based behavior change interventions selected to represent a range of countries, professional backgrounds, and academic disciplines.

### Recruitment

An invitation email describing the study was sent to (i) those who had participated in BCT training (online BCT Taxonomy training [http://www.bct-taxonomy.com/; last accessed on October 30, 2018], in-person BCT training workshops, or the BCT Taxonomy v1 project (8; http://www.ucl.ac.uk/health-psychology/ bcttaxonomy; last accessed on October 30, 2018), (ii) members of the project’s International Advisory Board, and (iii) email lists maintained by scientific and professional societies and centers (University College London’s Centre for Behavior Change, the Special Interest Group of the Society of Behavioral Medicine, European Health Psychology Society, United Kingdom Society for Behavioral Medicine, and Division of Health Psychology of the British Psychological Society). Using a “snowballing” method, those recruited were asked to recommend other experts for recruitment into the study.

Those expressing interest in becoming an expert judge (*n* = 227) completed a self-assessment questionnaire (see Appendix A in Supplementary Material) to evaluate their experience and expertise in behavior change interventions. To be eligible to participate in the study, experts needed to both (i) rate their expertise as ≥4 (on a 7-point scale, where 0 indicates “No expertise” and 7 indicates “Profound Expertise”) in BCTs, behavior change theories, and behavior change interventions; and (ii) report having some experience designing or helping to design behavior change intervention(s) that “used specific BCTs” and “was specifically grounded in behavior change theory/theories.” Based on these inclusion criteria, 123/227 (54.2 per cent) of the interested participants were eligible. We sent these 123 experts a second questionnaire, seeking information to help recruit experts across a range of countries, professional backgrounds, and academic disciplines (see Appendices B and C in Supplementary Material).

**Fig. 1.**
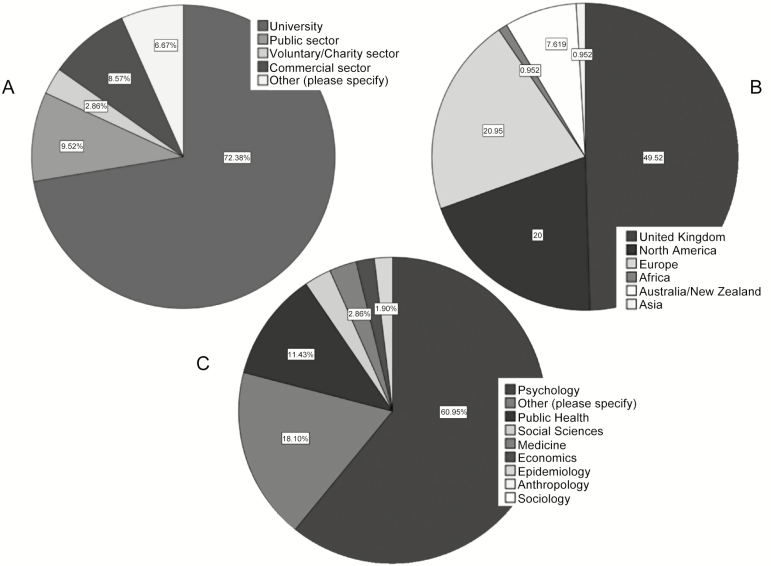
Descriptive characteristics of experts included in the consensus exercise. (A) Self-reported work sector; (B) geographical location; (C) professional background. Axis labels are in descending order, and label the pie chart in a clockwise direction.

Our final sample included 105 experts which was sufficient to provide task subgroups of at least 20 experts—a number found in previous work to demonstrate stability of consensus [24]. Nearly 50 per cent of experts were from the UK, 20 per cent were from other countries in Europe, 20 per cent from North America, and 10 per cent of experts were from Africa and Australia/New Zealand. Most experts worked in a university setting (75 per cent), in the field of psychology (60 per cent). Additional descriptive information about the experts’ backgrounds is depicted in Fig. 1, and information about the experts’ self-rated expertise in behavior change theory, interventions, and techniques is located in Appendix C in Supplementary Material.

### Procedure

Expert ratings for Rounds 1 and 3 were given via Qualtrics [25], a web-based software for administering surveys; the expert discussion in Round 2 was managed via the online forum “Loomio” [26]. In Rounds 1 and 3, experts rated links between a discrete set of BCTs and MoAs. To reduce participant burden, we limited the number of BCTs and MoAs included in the study based on the following criteria: (a) BCTs had to be commonly used within the intervention literature; therefore, we selected only those BCTs identified more than twice (*n* = 61) in a set of 40 systematically identified and coded intervention descriptions covering a range of different behaviors [27]; (b) MoAs were restricted to those contained within (i) the 14 theoretical domains described in the Theoretical Domains Framework [28] and (ii) the 12 most frequently occurring MoAs (which did not overlap with the Theoretical Domains Framework) identified in a systematic review of 83 behavior change theories [29]. This resulted in 61 BCTs and 26 MoAs. A full list of the MoAs and their definitions is provided in Table 1. To ensure the task was manageable for experts, we divided the BCTs into five groups and allocated either 13 or 14 BCTs × 26 MoAs (i.e., 338 or 364 possible links) for judgement by each group of experts. We block-randomized the 105 experts to one of five groups, with 21 experts per group, distributing experts from different countries, professional backgrounds, and academic disciplines among the five groups. To reduce possible bias in group ratings due to varying levels of familiarity with BCTs, BCTs were ordered according to the frequency with which they were used in interventions [8] and allocated to each of the five expert groups through stratified random allocation. Information about which BCTs were rated by each of the five groups is in Appendix D in Supplementary Material.

**Table 1:** List of 26 mechanisms of action rated for links with behavior change techniques

Mechanism label	Mechanism definition
Knowledge	An awareness of the existence of something
Skills	An ability or proficiency acquired through practice
Social/Professional Role and Identity	A coherent set of behaviors and displayed personal qualities of an individual in a social or work setting
Beliefs about Capabilities	Beliefs about one’s ability to successfully carry out a behavior
Optimism	Confidence that things will happen for the best or that desired goals will be attained
Beliefs about Consequences	Beliefs about the consequences of a behavior (i.e., perceptions about what will be achieved and/ or lost by undertaking a behavior, as well as the probability that a behavior will lead to a specific outcome)
Reinforcement	Processes by which the frequency or probability of a response is increased through a dependent relationship or contingency with a stimulus or circumstance
Intentions	A conscious decision to perform a behavior or a resolve to act in a certain way
Goals	Mental representations of outcomes or end states that an individual wants to achieve
Memory, Attention, and Decision Processes	Ability to retain information, focus on aspects of the environment, and choose between two or more alternatives
Environmental Context and Resources	Aspects of a person’s situation or environment that discourage or encourage the behavior
Social Influences	Those interpersonal processes that can cause oneself to change one’s thoughts, feelings, or behaviors
Emotion	A complex reaction pattern involving experiential, behavioral, and physiological elements
Behavioral Regulation	Behavioral, cognitive, and/or emotional skills for managing or changing behavior
Norms	The attitudes held and behaviors exhibited by other people within a social group
Subjective Norms	One’s perceptions of what most other people within a social group believe and do
Attitude towards the Behavior	The general evaluations of the behavior on a scale ranging from negative to positive
Motivation	Processes relating to the impetus that gives purpose or direction to behavior and operates at a conscious or unconscious level
Self-image	One’s conception and evaluation of oneself, including psychological and physical characteristics, qualities, and skills
Needs	Deficit of something required for survival, well-being, or personal fulfilment
Values	Moral, social or aesthetic principles accepted by an individual or society as a guide to what is good, desirable, or important
Feedback Processes	Processes through which current behavior is compared against a particular standard
Social Learning/Imitation	A process by which thoughts, feelings, and motivational states observed in others are internalized and replicated without the need for conscious awareness
Behavioral Cueing	Processes by which behavior is triggered from either the external environment, the performance of another behavior, or from ideas appearing in consciousness
General Attitudes/Beliefs	Evaluations of an object, person, group, issue, or concept on a scale ranging from negative to positive
Perceived Susceptibility/Vulnerability	Perceptions of the likelihood that one is vulnerable to a threat

### Conduct of the Consensus Exercise

#### Round 1: Initial Ratings of BCT × MoA Links

The aim of Round 1 was to establish an initial level of consensus among experts for each BCT × MoA link. For each of the 13 or 14 assigned BCTs, experts responded to the question, “Does the Behaviour Change Technique [e.g., Goal Setting] change behavior through the MoA [e.g., beliefs about one’s ability to successfully carry out a behavior (Beliefs about Capabilities)]?” on a 5-point scale (Definitely No, Probably No, Don’t Know/Uncertain, Probably Yes, and Definitely Yes). For each BCT, the 26 MoAs were randomized to appear in a different order to avoid ordering effects.

#### Round 2: Discussion of Uncertain and Disagreed Links

The aim of Round 2 was to facilitate a discussion of experts’ ratings of BCT × MoA links, particularly for the links that elicited high levels of disagreement and/or uncertainty among each of the five groups of experts in Round 1. Round 2 involved an online, anonymous, asynchronous (i.e., experts could contribute at a time of their choosing) discussion hosted via the digital discussion platform *Loomio* [26]. Experts were prompted to discuss (i) the 10 links that were rated “Don’t Know/Uncertain” by the highest number of experts within their group of experts, and (ii) the 10 for which there were nearly equivalent proportions of experts rating “Definitely No” and “Definitely Yes.” Prompts about these “uncertain” and “disagreed” links were included to maximize the usefulness of this round in moving the experts towards consensus; however, they were also given the opportunity to discuss other links and to explore their views on the task more generally.

During the Round 2 discussion, each expert was assigned an identification code to use as a pseudonym throughout the discussion to ensure anonymity. To facilitate participation across time zones, experts were not required to participate in live discussions; instead, they were given a 2 week period to comment on discussion threads within their group. Anonymous discussion moderators from the research team addressed questions raised by experts and prompted discussion periodically during the 2 week period by summarizing key points from the discussion and by conducting informal polls of experts’ opinions during the discussions. Round 2 took place 1 week after experts received the statistical summaries from Round 1.

#### Round 3: Final Ratings of All BCT × MoA Links

The aim of Round 3 was to establish a final understanding of experts’ views on the BCT × MoA links. Following the discussion round, experts were invited to rate links between the BCTs and MoAs that they had rated in Round 1. In response to feedback from experts during the discussion round, the wording and response options were slightly modified from that used in Round 1 (see Round 1 description for the original question and scale). For the final round, experts rated links by answering the question, “When [BCT] works, does it work through changing [MoA definition (MoA label)]?” Experts responded with “Definitely Yes,” “Definitely No,” “Uncertain,” or “Possibly.”

### Materials

#### Round 1

Prior to the start of the first round, experts were emailed their set of 13–14 BCTs, the 26 MoAs including definitions of both, and guidelines for the task (see Appendix E in Supplementary Material). During Round 1, for each question, experts were provided with the BCT definition, the MoA definition, and a diagram depicting that a BCT influences a MoA, which in turn influences behavior change

#### Round 2

After completing Round 1, each expert received an email with a personalized statistical summary of the results of Round 1. This included frequency distributions of their group’s responses, which were depicted alongside their own responses for each BCT × MoA link (see Appendix F in Supplementary Material). To summarize Round 1 data in an accessible format, the response options were collapsed into “Yes” (Definitely and Probably Yes), “No” (Definitely and Probably No), and “Uncertain” (Don’t Know/Uncertain).

#### Round 3

During Round 3, experts had access to both their personalized statistical summaries from the Round 1 ratings and were provided transcripts of their group’s Round 2 discussion. The detailed information from the previous rounds allowed experts to re-evaluate their original ratings for each link, in light of the thoughts and ratings of the other experts in their group.

#### Procedures to Evaluate Effects of Group Membership

To detect any effect of group membership (i.e., whether certain groups of experts were more likely to rate BCT × MoA links in a particular way), two of the 13 or 14 BCTs rated by each expert group were rated by all of the experts in Round 1 (i.e., 52 shared BCT × MoA links were rated across the five groups). The BCTs rated by all experts were the two BCTs most frequently identified in our dataset of studies evaluating BCT v1 Taxonomy [8]: “Instruction on How to Perform the Behaviour” and “Social Support (Unspecified).”

To facilitate comparison of the discussions across groups, two links were discussed in Round 2 by all five expert groups, by selecting the two BCT × MoA links, out of a total of 52 shared BCT × MoA links, for which there was the most disagreement (BCT: Instruction on how to perform the behavior → MoA: Intention), and the most uncertainty (BCT: Social Support [Unspecified] → MoA: Attitude towards the Behaviour) across all five groups.

### Data Analysis

To address the research questions, through which MoAs do experts agree that BCTs influence behavior (Question 1), and through which MoAs do experts agree that BCTs do not influence behavior (Question 2), we conducted descriptive analyses (in MS Excel) on the final ratings from Round 3. This enabled us to describe where there was consensus on links between the 61 BCTs and 26 MoAs. Expert consensus was defined as more than 80 per cent of experts in agreement that a BCT was either definitely linked or definitely not linked to a MoA. To examine the specificity with which BCTs influence behavior, we evaluated the BCT × MoA links identified to be definitely linked, to determine whether BCTs were linked to one or more MoAs (Question 3), this information was further analyzed to determine whether all BCTs could be linked to one or multiple MoAs (Question 4), and whether all MoAs could be linked to one or multiple BCTs (Question 5). The distribution of the proportion of experts rating “definitely yes,” “definitely no,” “possibly,” and “don’t know/uncertain” was assessed to report results of disagreement and uncertainty about BCT × MoA links (Question 4).

The final (Round 3) ratings were represented visually in four heat maps generated in *R* [30]. A heat map is a visual representation of a data matrix—in this instance, the matrix of BCTs (rows) linked to MoAs (columns)—where the values in the cells are represented by colors, and shaded to indicate the strength or “heat” of that value. In this case, the values in the cells represent the percentage of experts who agree that a BCT and MoA are “definitely” linked. The darker the shading in the cells of the heat map, the larger the proportion of experts who rated the link as “definitely yes” linked, or “definitely no” not linked. The darkest shading in the cells represents 95%–100% of experts agreed on the link. The heat map clusters rows (BCTs) and columns (MoAs) by similarity, such that BCTs linked to similar MoAs are clustered together vertically, and MoAs linked to similar numbers of BCTs are clustered together horizontally. This clustering facilitates visual identification of patterns present within the data, but not statistical inference.

To examine any possible influences of group membership on ratings, intraclass correlation coefficients were calculated for experts’ ratings on the 2 BCTs × 26 MoAs considered by all experts (i.e., 52 links). These intraclass correlation coefficients were calculated for ratings in Round 1 (to examine influences of group allocation) and Round 3 (to examine changes following the group discussion round). The extent to which variance in the ratings can be attributed to group membership can be understood by translating the intraclass correlation coefficient value into a percentage, for example, if the intraclass correlation is .01, this means 1 per cent of the variance in the ratings can be attributed to group membership properties. In the absence of standard criteria for intraclass correlation values and group ratings, the results describe rather than evaluate the influence of group membership on the consensus within the group. The Round 1 intraclass correlation coefficients were predicted to be small, because experts were randomly assigned to groups, and stratified to represent different countries, professional backgrounds, and academic disciplines. Round 3 intraclass correlation coefficients were predicted to be larger due to the likely influence of group-specific discussion about the links.

## Results

### Round 1

All experts (*n* = 105) participated in Round 1. After Round 1, at least 80 per cent of experts agreed that 13 BCT × MoA links (0.81 per cent) were “definite” links, and 3 were “definitely not” links (0.19 per cent). At least 50 per cent of experts agreed that 83 BCT × MoA links (5 per cent) were “definite” links, 147 (9 per cent) were “possibly” links, 53 (3 per cent) were “possibly” not links, and 296 (19 per cent) were “definitely” not links. There were no links for which more than 50 per cent of experts were uncertain about the BCT × MoA link.

### Round 2

During Round 2, experts in five groups collectively discussed 102 links: the 10 links rated “Don’t Know/Uncertain” by the largest percentage of experts in their group, the 10 for which a nearly equal percentage of experts rated “Definitely No” and “Definitely Yes,” and two links which were discussed by all five groups, which were the most uncertain and disagreed links across all experts. Ninety-two of the 105 experts actively participated in the discussion round, with the number of comments per expert ranging from 1 to 40 (*M* = 13.96, *SD* = 7.103). The number of experts who did not participate within their discussion group ranged from 0 to 4 experts per group and the total number of comments within a discussion group ranged from 213 to 353 comments. There were no significant differences in the mean number of comments per expert across groups, *F* (4, 95) *=* 1.684, *p* = .161. The frequency with which the experts participated in the discussion round suggests that experts were engaged in the task, and the comments from experts during the round indicate that experts found the task helpful in reaching consensus. For example, comments included:

*“I put uncertain, as I too could not see how [Instruction on how to Perform the Behavior] would necessarily facilitate Intention to act as [Expert] points out… I think the example about smoking from [another Expert] illustrates when this would not apply very effectively. I would change my rating to ‘no’ now”*;

I have found this one of the most challenging to call and therefore waited to see the arguments of others as I was unable to decide a camp. I too do not feel that intention is the primary MoA [for BCT social comparison], but I see the argument put forward by [Expert]. […] However my hunch is still that this is not a key MoA therefore I will rate it as ‘no’

The pattern of ratings and feedback from experts indicated that experts had difficulty with the initial rating task, and in particular found it difficult to discern between “Possibly Yes” and “Possibly No.” Experts stated that if they could not judge a link as either definitely linked or not linked, it was difficult to discern which direction to judge the “possibility” of the link, and requested one “possibly” option instead of two. For example, one expert noted, *“The two [‘probably’] options created a lot of grey area, which experts interpreted differently.”* Experts also noted having difficulty linking BCTs in general with specific MoAs. For example, experts in two different groups commented on their difficulty rating links with intentions. One expert mentioned, *“In general, I struggled with INTENTION as a key MoA for most BCTs. I see Intention as so proximal to behavior (and analogous to overall motivation) that it is almost always a result of other (more critical) MoAs, no matter which theoretical perspective one adopts,”* and an expert in a different group made a similar comment, “*While I believe ‘intention’ is not a key MoA in this case, and am thus happy to keep my no vote, I also struggled with ‘intention’ as MoA in general - I thought it would be involved but not necessarily key to achieving change for almost all BCTs.”* Some experts also noted that it was challenging to make singular links between BCTs and specific MoAs, *“It is very difficult to think about the individual BCTs in isolation. My brain is forced to think about models of behaviour change, directions of causality and other BCTs before making a decision ‘yes’, ‘no’ or ‘unsure’. Although I might say ‘yes’ to a specific BCT, it’s likely that what I’m really saying is that the BCT in question is part of a cluster, but probably plays the biggest part in that cluster.”*

### Round 3

Nearly all experts (*n* = 100, 95 per cent) participated in the final ratings round. Of the 1,586 possible links (61 BCTs × 26 MoAs), consensus was reached for 90 BCT × MoA links as “definite” links (Question 1) and 464 as “definitely not” links (Question 2; see Appendix G in Supplementary Material). Of the 102 links for which there had been high disagreement and/or uncertainty and were discussed in Round 2, expert consensus emerged for eight links in Round 3.

**Fig. 2.**
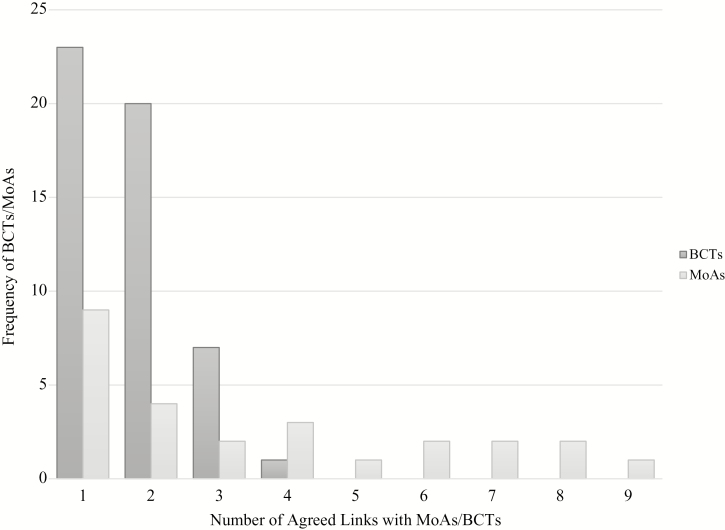
The frequency with which behavior change techniques (BCTs) were definitely linked to mechanisms of action (MoAs) by ≥80% of experts is depicted in the dark grey bars, and the frequency with which MoAs were linked to BCTs by ≥80% of experts is depicted in the light grey bars.

Of the 61 BCTs, 51 were rated by experts as definitely linked to at least one MoA. Of the 26 MoAs, 21 were definitely linked to at least one BCT (Questions 5 and 6). Figure 2 depicts the specificity with which BCTs link to MoAs, with frequencies included for the number of BCTs with one or more “definitely” linked MoAs, and the number of MoAs with one or more “definitely” linked BCTs. Twenty-three BCTs were linked to only one MoA, and 20 BCTs were linked to only two MoAs (Question 3). MoAs were linked to 1–9 BCTs, with the MoA Motivation linked to nine different BCTs. The 10 BCTs with no definite links to MoAs were as follows: Action Planning, Monitoring of Behavior by Others without Feedback, Monitoring of Outcomes of Behavior without Feedback, Behavioral Substitution, Generalization of the Target Behaviour, Credible Source, Non-Specific Incentive, Pharmacological Support, Body Changes, and Self-Talk. The five MoAs with no definite links to BCTs were as follows: General Attitudes and Beliefs, Needs, Optimism, Social Professional Role and Identity, and Values.

**Fig. 3.**
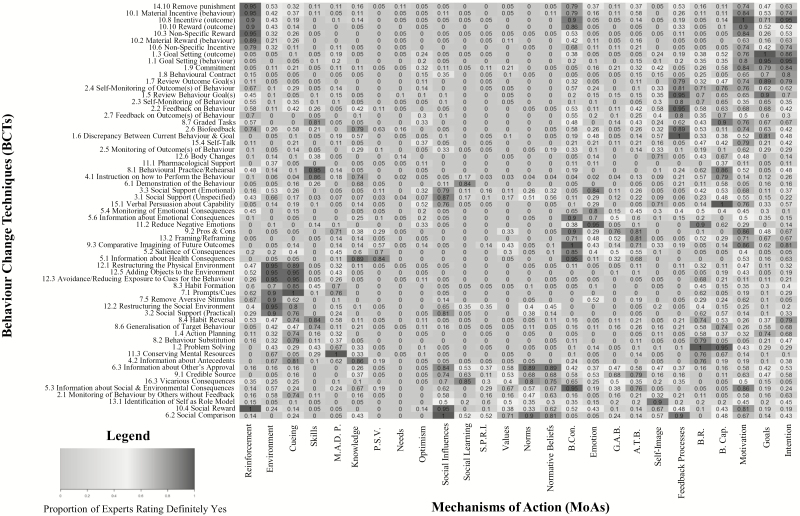
A heat map indicating the proportion of experts rating a behavior change technique (BCT) was “definitely” linked to a mechanism of action (MoA). Values range from 0 to 1, with values closer to 1 shaded in the darkest grey. A 1 indicates 100% of experts agreed a BCT that was definitely linked to a MoA. M.A.D.P. = Memory, Attention, and Decision Processes; P.S.V. = Perceived Susceptibility and Vulnerability; S.P.R.I = Social/Professional Role and Identity; B. Con. = Beliefs about Consequences; G.A.B. = General Attitudes and Beliefs; A.T.B = Attitude towards the Behavior; B.R. = Behavioral Regulation; B.Cap. = Beliefs about Capabilities.

**Fig. 4.**
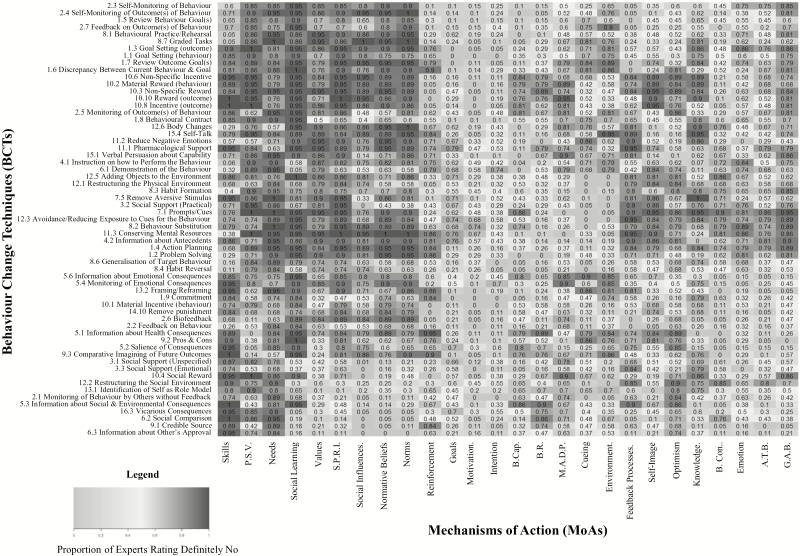
A heat map indicating the proportion of experts rating a behavior change technique (BCT) was “definitely” not linked to a mechanism of action (MoA). Values range from 0 to 1, with values closer to 1 shaded in the darkest grey. A 1 indicates 100% of experts agreed a BCT was definitely not linked to a MoA. M.A.D.P. = Memory, Attention, and Decision Processes; P.S.V. = Perceived Susceptibility and Vulnerability; S.P.R.I = Social/Professional Role and Identity; B. Con. = Beliefs about Consequences; G.A.B. = General Attitudes and Beliefs; A.T.B = Attitude towards the Behavior; B.R. = Behavioral Regulation; B.Cap. = Beliefs about Capabilities.

Two heat maps (Figs. 3 and 4) present expert responses (i.e., Definitely Yes, or Definitely No) to the question, “When BCT X works, it does so by changing MoA Y.” All of the values in the heat map, which represent the proportion of experts who agreed on a link as either “definitely yes” linked, or “definitely no” not linked, can also be viewed in table format in Appendix G in Supplementary Material. The utility of the heat map is that patterns can be identified visually across a very large data set such that BCTs which experts agreed were linked to similar MoAs are positioned closer together in the heat map, and MoAs which are linked to a similar frequency of BCTs are closer together.

There were 1,032 (65 per cent) BCT × MoA links which did not meet the consensus criterion. For 163 links, this was due to strong disagreement among the experts (i.e., one-third of experts said “Definitely Yes,” one-third said “Definitely No,” and one-third said either “Possibly” or “Don’t Know/Uncertain”; Question 4). For an additional set of 340 links, there was generally agreement among the experts, but not enough to meet the prespecified consensus criterion. Specifically, 50%–80% of experts rated 255 and 85 links as “Definitely” not links and definitely links (respectively), with the remaining experts providing ratings of “possibly” and “Don’t know/Uncertain.” For the remaining 529 links, no meaningful trends emerged among the response options selected by experts.

### Analysis of Between-Group Differences in Rating Patterns (Intraclass Correlation Coefficients)

To assess whether there were group differences in expert ratings, we examined intraclass correlation coefficients for the two BCTs shared across groups (i.e., Instruction about how to perform the behavior; Social Support [Unspecified]). In Round 1, the intraclass correlation coefficients were small for all BCT × MoA links (*|range|=* 0.00 − 0.10), suggesting that allocation to groups did not affect ratings (Table 2). As anticipated, intraclass correlation coefficients increased from Round 1 to Round 3 for 37 BCT × MoA links, reflecting an increase in agreement within the groups after the discussion in Round 2. In Round 3, 1 out of 52 (1.9 per cent) intraclass correlation coefficients was large (for the MoA “General attitudes and Beliefs” with the BCT “Social support (unspecified)”), and 13 of 52 (25 per cent) were moderately sized. Ten of those were for the BCT “Social support (unspecified)”; it thus seems that the Round 2 discussions led to higher agreement on the meaning of this BCT in some groups than in others.

**Table 2:** Intraclass correlation coefficients for the BCT–MoA links rated by all experts

	Social Support (Unspecified)			Instruction on how to Perform the Behaviour		
Mechanism of Action	Round 1	Round 3	∆	Round 1	Round 3	∆
Knowledge	0.05	−0.01	−0.06	−0.03	−0.04	−0.01
Skills	0.05	0.07	0.02	0.00	0.02	0.02
Behavioral Regulation	0.07	0.12	0.05	0.09	0.11	0.02
Social Influences	0.00	−0.01	−0.01	−0.01	0.01	0.02
Memory, Attention, Decision Processes	0.01	0.02	0.01	0.04	0.02	−0.02
Social Professional Role / Identity	0.02	0.10	0.08	0.01	0.02	0.01
Beliefs about Capabilities	0.09	0.18	0.09	0.02	0.01	−0.01
Beliefs about Consequences	0.03	0.05	0.02	−0.03	0.01	0.04
Optimism	−0.04	0.01	0.05	−0.02	0.01	0.03
Intention	0.00	0.10	0.10	−0.02	0.08	0.10
Goals	0.04	0.07	0.03	0.07	0.06	−0.01
Reinforcement	0.10	0.13	0.03	0.03	0.04	0.01
Emotion	0.03	0.08	0.05	0.00	0.03	0.03
Environment	0.01	0.01	0.00	0.03	0.00	−0.03
Norms	−0.01	−0.02	−0.01	0.00	0.01	0.01
Subjective Norms	0.00	−0.03	−0.03	−0.02	0.03	0.05
Attitude towards the Behavior	0.01	0.24	0.23	−0.01	0.23	0.24
Motivation	0.03	0.07	0.04	0.00	0.00	0.00
Self-image	−0.03	0.00	0.03	0.04	0.07	0.03
Needs	−0.02	0.09	0.11	−0.03	0.05	0.08
Values	−0.02	0.17	0.19	0.00	0.06	0.06
Feedback Processes	−0.01	0.02	0.03	0.00	0.02	0.02
General Attitudes and Beliefs	0.08	0.26	0.18	0.03	0.10	0.07
Social Learning	0.04	0.21	0.17	0.07	0.05	−0.02
Cueing	0.07	0.06	−0.01	−0.02	0.05	0.07
Perceived Susceptibility/Vulnerability	0.03	0.03	0.00	0.05	0.05	0.00

*∆ =* Change in the intraclass correlation coefficient from Round 1 to Round 3.

‘Social Support (Unspecified)’ and ‘Instruction on how to Perform the Behaviour’ are the Behaviour Change Techniques (BCTs) rated by all experts for links with the 26 mechanisms of action. Values in the table indicate the intraclass correlation coefficient for each link rated by all experts who had been randomly allocated to one of five groups.

## Discussion

The aim of the current study was to identify links between 61 commonly used BCTs, and 26 frequently occurring MoAs. The experts reached consensus for 51 out of 61 BCTs and 20 out of 26 MoAs. Twenty-three of these 51 BCTs were linked to one MoA, and 20 BCTs to two or more MoAs, with a total of 90 identified links. Experts also agreed that 464 out of a total of 1,586 possible links definitely did not exist—agreeing these should not be targeted in interventions. Experts did not reach agreement on the remaining 1,032 BCT × MoA links, suggesting disagreement on the majority of BCT × MoA links. No links were identified (either way) for 10 frequently used BCTs and 6 MoAs. Taken together, this study has identified 51 potentially effective BCTs for modifying 20 frequently used MoAs to inform intervention development and evidence synthesis. Additionally, this study identified which BCT × MoA links are likely to not exist, and revealed that on the majority of links experts could not achieve 80 per cent agreement. These links could be of particular interest for future research.

Based on the pattern of BCT × MoA links which were either definitely agreed upon, or which showed a trend towards agreement but failed to reach consensus, experts agreed that most of the BCTs assessed in this task change behavior through changes in motivation and intention. There were also a large number of BCTs that were hypothesized to operate through the MoAs: beliefs about capabilities, beliefs about consequences, and behavioral regulation. BCTs linked to reinforcement, cueing, and environmental context and resources were agreed by nearly all experts rating the links. One possible explanation for the strength of the evidence for these links could be the extent to which the theoretical literature explicitly describes techniques for changing these MoAs. Similarly, the BCT v1 Taxonomy is structured hierarchically to group together BCTs which are more similar in function [7], and several of these groupings appear within the heat map (in terms of vertical proximity) as a result of the consensus among the experts as to how individual BCTs link to individual MoAs, which could indicate shared theoretical hypotheses across different consensus studies.

For some of the most frequently used BCTs (e.g., Action Planning, Credible Source, and Behavioral Substitution [26]), there was no consensus regarding the MoAs they target. Given the engagement of experts with the task, and the increase in agreement across rounds, it is possible additional consensus rounds, more participants per group, and/or an additional consensus exercise with different experts could increase the number of agreed BCT × MoA links. However, the lack of agreed links may be because these BCTs are linked to different MoAs in several different theories, and possibly only work in combination with other BCTs—making it difficult to judge these links in isolation. Furthermore, there are several MoAs which occur frequently within theories of behavior change (e.g., Needs, Values, and Optimism), but for which experts could not come to consensus regarding the BCTs that are able to elicit change in them. One possibility is these MoAs need to be targeted by a variety, or a group of BCTs, as noted by experts during the discussion round.

A BCT–MoA–behavior change effect depends on both the effect of the BCT on the MoA and the effect of the MoA on behavior change. Expert ratings may have been influenced by beliefs about both of these effects. For example, nonlinks could emerge even if the experts had confidence in the BCT’s ability to alter the MoA, but had limited confidence in the link between the MoA and behavior change. This concern was raised by experts during the discussion round. The present study cannot determine whether nonlinks were a result of experts’ confidence in the ability to alter the MoA, or the influence of the MoA on behavior change. However, the comments by experts prompted the modification of the question wording in Round 3 such that experts considered their judgments in light of the hypothetical, “When this BCT works….” Further research is needed to explore on what basis experts are making their judgments. For example, 95 per cent of experts agree that “Goal Setting (behavior)” changes behavior by eliciting changes in intentions; yet, experts commented (in Round 2) on their difficulty linking any BCTs to intention. This may be due to research suggesting that changes in intentions lead to small- to medium-sized changes in behavior [31–34].

We chose a stringent consensus criterion of 80 per cent agreement among experts in this study. In a systematic review of previous expert consensus studies, the most commonly used method for assessing consensus is percentage of experts in agreement, and the median threshold value used was 75 per cent, with a range of 50%–97% [35]. We chose a stringent criterion because of the relatively small number of experts rating each link compared with the number of links rated by each expert. However, the links for which more than two-thirds of experts agreed (i.e., the more moderately shaded links in the heat map) may also be considered for hypothesis testing.

At present, intervention developers tend to consult theory, empirical literature, and common sense to decide which BCTs to utilize for modifying the MoA deemed relevant for changing the behavior of interest. Hence, these links are based on the interpretation of the literature by that individual (or research team), which—as this study suggests—may be quite different from how other researchers interpret the literature. The current data provide evidence about the shared judgements of experts and is a resource that can be drawn upon for intervention development and generate data-driven hypotheses. The heat maps, and list of agreed links and nonlinks, can be used to select BCTs to target relevant MoAs to change behavior. Similarly, these data can be used to determine which MoAs should be measured to evaluate the process of change within an intervention, and/or to inform intervention evaluation. Of additional interest to intervention designers and evaluators are the data indicating which BCTs are not linked to specific MoAs, and which MoAs are not linked to specific BCTs.

Furthermore, these results generate hypotheses about effective links and provide the basis for a program of empirical research to test the most promising BCT–MoA links. The varying levels of agreement among 100 experts, for approximately 1000 BCT × MoA links, provide an important foundation for future empirical testing, and to increase our understanding of how intervention components have their effects.

### Limitations

The range of links identified in this study was restricted to those we evaluated, which represented only a subset of possible links (i.e., 61 of 93 BCTs in the BCT v1 Taxonomy). Secondly, we chose specific inclusion/exclusion criteria for selecting experts, and it is possible a different recruitment strategy might have led to different results; however, the large number of experts included in the study, the screening tools used to balance the pool of experts, and the variety of recruitment procedures used were intended to mitigate bias in the findings due to the expert pool. There was some evidence that certain expert groups were able to agree more upon certain BCT × MoA links than other groups, based on the between-group analyses. Most of the links with better agreement among some expert groups than others were for the BCT “Social support (unspecified),” which in previous studies has lower-than-average reliability [7, 36]. The question prompt and rating scale for evaluating BCT × MoA links changed from Round 1 to Round 3, which could have limited the consensus among experts. The use of the original rating scale may have reduced the total number of links agreed upon at the end of the exercise due to the difficulty experts reported after Round 1. Lastly, BCTs were rated on the extent to which they change behavior through a finite set of MoAs. These MoAs were selected because of their frequency in behavior change theories; there may be other important MoAs to consider which were not captured.

### Future Directions

Although the current research addresses theoretically based MoAs, these MoAs have not been linked to theories or theoretical frameworks directly. Theories propose how MoAs interact to have an effect on behavior and therefore how and why the effects of BCTs might occur. There is clearly scope to explore whether the BCT × MoA links have additive (independent), synergistic, or antagonistic effects, as has been investigated in previous research [37–39].

To improve theory development, other work in the current program of research (Michie et al.) is assessing whether the agreed links between BCTs and MoAs are consistent with the BCT-MoA links that are specified in behavior change theories. For more information about the progress of this work, and to access the latest evidence on BCT × MoA links, as well as a link to view a high resolution, color version of the heat maps, see https://theoryandtechniquetool.humanbehaviourchange.org/; last accessed on October 30, 2018. The next stage in this program is to examine how the links found in this study relate to a previous study (see Carey et al., under review), examining links found in the published behavior change intervention literature and to investigate differences in the links found in the two studies.

## Conclusions

The findings from this study represent a systematically drawn consensus of experts’ judgments about the mechanisms through which BCTs do or do not change behavior. The definite links between BCTs and MoAs identified in this study—90 present and 464 not present—can be used to inform intervention development and synthesis. The considerable uncertainty about the majority of BCT-MoA links could be of particular interest for future studies. These results can be considered as a first level of evidence, generating hypotheses which can be confirmed or refuted through further empirical studies to understand the mechanisms by which BCTs have their effects in changing behavior.

## Supplementary Material

kay082_suppl_Supplementary_Material
