# Supplementary material for: Links Between Behavior Change Techniques and Mechanisms of Action: An Expert Consensus Study
Source: Ann Behav Med. 2018 Nov 19;53(8):708–20. doi: 10.1093/abm/kay082 (PMC6636885; doi:10.1093/abm/kay082)
Supplement: kay082_suppl_Supplementary_Material [file kay082_suppl_supplementary_material.docx]

**Supplementary Materials**

Appendix A

Study 2 Expert Agreed Explicit Links: Expert Recruitment Self-Evaluation Form

1. **To what extent have you:**

| 1. Designed or helped to design a behaviour change intervention(s) that used specific behaviour change techniques (BCTs)?   Extensively  To Some Extent  Not at all | | | | | | | | | |  |
| --- | --- | --- | --- | --- | --- | --- | --- | --- | --- | --- |
| 1. Used a taxonomy of BCTs (e.g. BCT Taxonomy v1) to code, design, or evaluate a behaviour change intervention?   Extensively  To Some Extent  Not at all | | | | | | | | | |  |
| 1. Designed or helped to design a behaviour change intervention(s) that was specifically grounded in a behaviour change theory/theories?   Extensively  To Some Extent  Not at all | | | | | | | | | |  |
| 1. Published papers/manuals/protocols of interventions that specify component BCTs?   Extensively  To Some Extent  Not at all | | | | | | | | | |  |
| 1. Published papers/manuals/protocols of interventions that specify behaviour change theory/theories?   Extensively  To Some Extent  Not at all | | | | | | | | | |  |
| 1. Undertaken a narrative or systematic review of behaviour change literature specifying interventions in terms of BCTs?   Extensively  To Some Extent  Not at all | | | | | | | | | |  |
| 1. Undertaken a narrative or systematic review of behaviour change literature specifying interventions in terms of behaviour change theory?   Extensively  To Some Extent  Not at all | | | | | | | | | |  |
| \| 1. **For approximately how many hours have you used BCT methodology?**   **(e.g. 0-5, 10-20, 25-50, 50-100, 100+)** \|  \| \| --- \| --- \| \| 1. **For how many studies have you used BCT methodology?** \|  \| \| 1. **For how many studies have you explicitly used behaviour change theory?** \|  \|  1. **How would you rate your knowledge/expertise in the following?**   **(where 0 = no knowledge/expertise and 7 = profound knowledge/expertise):** | | | | | | | | | | |
| Behaviour change theories | | 0 | 1 | 2 | 3 | 4 | 5 | 6 | 7 | |
| Behaviour change techniques | | 0 |  | 2 | 3 | 4 | 5 | 6 | 7 | |
| Behaviour change interventions (evaluation or design) | | 0 | 1 | 2 | 3 | 4 | 5 | 6 | 7 | |
| 1. **Other relevant experience (please specify):** | | | | | | | | | | |
| **7. Please provide your contact details:** | | | | | | | | | | |
| **Name:** |  | | | | | | | | | |
| **Email:** |  | | | | | | | | | |

Appendix B

Self-rated Expertise in Designing or Helping Design Behaviour Change Interventions Using Specific Behaviour Change Techniques and/or Theory

*Note.* Values indicate the number of experts who rated the extent to which they had designed or helped design a behaviour change intervention using specific behaviour change techniques, or behaviour change theory (See also Appendix A; Questions 1a and 1c).

Appendix C

Self-rated Expertise in Behaviour Change Theories, Behaviour Change Techniques, and Behaviour Change Interventions

*Note.* Values indicate the number of experts who rated the extent of their expertise in behaviour change theories, behaviour change techniques, and behaviour change interventions (See Appendix A; Questions 5a-5c).

Appendix D

Behaviour Change Techniques Rated by Each Expert Group

| Group A | Group B | Group C | Group D | Group E |
| --- | --- | --- | --- | --- |
| 1.1 Goal Setting (Behaviour) | 5.1 Information about the health consequences | 3.2 Social support (practical) | 9.1 Credible source | 5.3 information about social & environmental consequences |
| 2.7 Feedback on outcome(s) of behaviour | 1.4 Action Planning | 12.5 Adding objects To The Environment | 2.2 Feedback on behaviour | 1.2 Problem Solving |
| 2.3 Self-monitoring of behaviour | 6.1 Demonstration of the behaviour | 7.1 Prompts/cues | 2.1 Monitoring of behaviour by others without feedback | 1.3 goal setting (outcome) |
| 1.5 Review behaviour goal(s) | 1.6 Review outcome goal(s) | 8.1 Behavioural practice/rehearsal | 12.1 Restructuring the physical environment | Monitoring of outcome of behaviour without feedback |
| 8.3 Habit Formation | 11.1 Pharmacological support | 2.4 Self-monitoring of outcome(s) of behaviour | 3.3 Social Support (Emotional) | 4.2 Information About Antecedents |
| 5.2 Salience of consequences | 10.2 Material reward (behaviour) | 6.1 Social comparison | 2.6 Biofeedback | 1.6 discrepancy between current behaviour & goal |
| 12.2 Restructuring the social environment | 10.3 Non-specific reward | 13.2 Framing/reframing | 10.1 Material incentive (behaviour) | 15.1 Verbal persuasion about capability |
| 1.8 Behavioural Contract | 10.6 Non-specific incentive | 11.2 reduce negative emotions | 1.9 Commitment | 10.4 Social reward |
| 5.6 Information about emotional consequences | 8.2 Behaviour Substitution | 9.2 Pros & cons | 6.3 Information about others' approval | 11.3 Conserving mental resources |
| 5.4 Monitoring of emotional consequences | 15.4 Self-Talk | 7.5 Remove aversive stimulus | 8.6 Generalisation of target behaviour | 10.8 Incentive (outcome) |
| 13.1 Identification of self as role model | 12.3 Avoidance/Reducing exposure to cues for the behaviour | 8.7 Graded tasks | 8.4 Habit reversal | 10.10 Reward (outcome) |
| 16.3 Vicarious Consequences |  | 12.6 Body changes | 14.10 Remove punishment | 9.3 Comparative imagining of future outcomes |

*Note.* All expert groups rated Instruction on How to Perform the Behavior, and Social Support (Unspecified).

Appendix E

Content of the Guidelines for Round 1 of the Consensus Exercise

**Aims of Study:** This study aims to develop a shared understanding of behaviour change techniques (BCTs) and the mechanisms of action through which they influence behaviour. A summary of the project is appended to this document (Appendix A).

**Who are the participants?** You are one of 105 experts from 18 countries, selected because you have a high level of expertise in theories and techniques of behaviour change.

**What are we asking you to do?** The consensus exercise is a modified Nominal Group Technique (NGT) (23), involving 3 rounds. In Round 1, you will be directed to an online questionnaire. You will be presented with 13 BCTs, one at a time, and asked to consider whether each BCT changes behaviour through any of a given set of 26 mechanisms of action. Thus, you will be asked to respond to 13 sets of 26 questions, where each set relates to 1 BCT × 26 mechanisms. You will be prompted to take a break after completing each set – you may complete Round 1 in one sitting or several.

**What is a Behaviour Change Technique (BCT)?** BCTs are the potentially active ingredients of behaviour change interventions. For this study, BCTs will be taken from the 93-item BCT Taxonomy Version 1 (BCTTv1; http://www.ncbi.nlm.nih.gov/pubmed/23512568)

**What is a Mechanism of Action?** These are defined as “the processes through which a BCT affects behaviour”. The following diagram may be helpful in conceptualising the processes through which a particular BCT might affect behaviour:


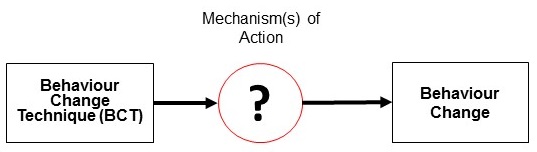


**Notes:**

- - - 1. We are not evaluating the effectiveness of BCTs in this study (i.e. how likely it is that a particular BCT changes behaviour). When rating BCT-mechanism links (see example below), please focus on the mechanisms you believe a BCT might change, in order to change behaviour.
      2. There are many mechanisms of action through which a BCT might affect behaviour. When considering whether a given BCT affects behaviour through a particular mechanism, you may draw on evidence, theory and/or experience.
      3. In order to ensure consistency across experts, and with the aim of developing a shared understanding of BCT-mechanism links, it is very important that you base your answers on the definitions, rather than labels, of the BCTs and mechanisms. Definitions for all BCTs and mechanisms will appear on screen during the consensus exercise, and these can also be found in Appendices B and C. Please read and re-read these definitions before beginning the task.

**Sample Question:**

Does the Behaviour Change Technique Goal Setting (Behaviour) change behaviour through the mechanism: An awareness of the existence of something (knowledge)?

 Definitely No

 Probably No

 Uncertain / Don’t Know

 Probably Yes

 Definitely Yes

*Note.* Additional information provided in the guidelines document was excluded from this paper, this information included a project summary, BCT definitions, and mechanism of action definitions. For more information about the project see the project protocol, published in *Annals of Behavioral Medicine,* for BCT definitions see the BCT taxonomy app or website: www.bcts.23.co.uk, and for more information about the mechanisms of action included in this study, see the above Table 1.

Appendix F

Sample Statistical Summary Sent to Experts after the Completion of Round 1


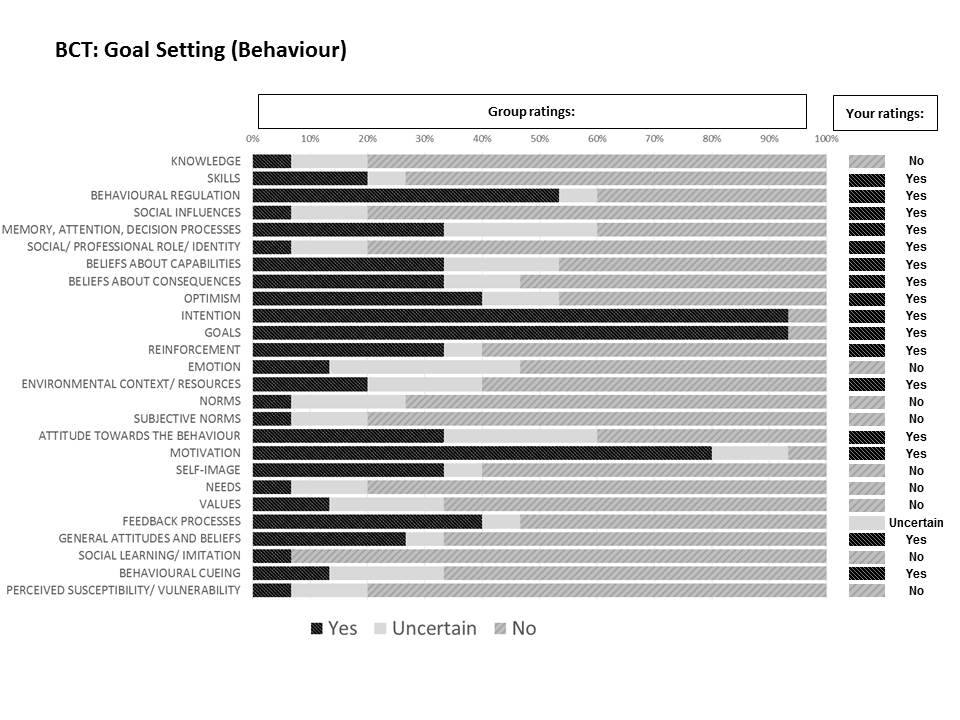


*Note.* The above distribution depicts the frequency with which experts thought the BCT ‘Goal Setting (Behaviour) was linked to any of the 26 mechanisms of action. For Round 1, Yes = Definitely Yes + Probably Yes, No = Definitely No + Probably No.
